# Editorial: 5-HT_2A/2B/2C_ Receptors, Memory, and Neuropsychiatric Disorders

**DOI:** 10.3389/fphar.2016.00009

**Published:** 2016-02-01

**Authors:** Antonella Gasbarri, Bettina Bert, Alfredo Meneses

**Affiliations:** ^1^Department of Applied Clinical and Biotechnologic Sciences, University of L'AquilaL'Aquila, Italy; ^2^Department of Veterinary Medicine, Institute of Pharmacology and Toxicology, Freie Universität BerlinBerlin, Germany; ^3^Departamento de Farmacobiología, Centro de Investigación y de Estudios Avanzados del Instituto Politécnico NacionalMexico City, Mexico

**Keywords:** 5-HT_2A_ receptor, 5-HT_2B_ receptor, 5-HT_2C_ receptors, memory disorders

It is very well known that memory formation and forgetting represent important cognitive functions modulated by several neurotransmitters, including serotonin (5-hydroxytryptamine, 5-HT) (e.g., Fioravanti and Di Cesare, 1992; Wagner and Davachi, 2001; Wixted, 2004; Mansuy, 2005; Hardt et al., 2013; Hupbach; Callaghan et al., 2014; Li et al., 2015; Meneses), and memory dysfunction is present in several neuropsychiatric disorders (Meyer-Lindenberg et al., 2012; Millan et al., 2012, 2014). How does the brain become dysfunctional? Such a question has intrigued neuroscientists for many years. Over recent decades, neuroscience research has shed light on this complex issue and growing evidence is providing important insights. For instance, several lines of evidence demonstrate that dysfunctions in memory processes occur in several neuropsychiatric disorders, including Alzheimer's disease (AD), characterized by memory deficits and dementia; however, dysfunctional memory is observed in other age-related neurodegenerative disorders, such as schizophrenia, post-traumatic stress disorder, stroke, etc. (Millan et al., 2014; Hashimoto, 2015). So far, no effective treatment for dysfunctional memory exists (e.g., Millan et al., 2012, 2014; Sun et al., 2015); this area is hampered by the absence of neural markers associated to memory. Hence, progress in the fields relating to memory, amnesia, forgetting (e.g., Tellez et al., 2012) and AD (e.g., McConathy and Sheline, 2015; Muenchhoff et al., 2015; Scarr et al., 2015) as well as mild cognitive impairment (Eshkoor et al., 2015) would be facilitated considerably by identification of suitable neural markers. Serotonin is involved in several physiological and pathophysiological processes in mammals and in the treatment of psychiatric disorders (Meneses). It is very well known that 5-HT receptor heterogeneity allows for greater advances, and 5-HT _2A∕2B∕2C_ receptors are emerging as important tools for preclinical and clinical investigation. This special issue provides a deep overview of important advances on cerebral modifications associated with neuropsychiatric alterations, including dysfunctional memory. In particular, some papers analyse the role of serotonin and dopamine receptors in motivational and cognitive disturbances of schizophrenia (Sumiyoshi et al.), while others are focused on the missing link of the central serotonin-2A 5-HT_2A_ receptor dysfunction in depression and epilepsy (Guiard and Di Giovanni) and on the activity-regulated cytoskeleton-associated protein's putative role in regulating dendritic plasticity, cognitive processes, and mood in animal models of depression is presented (Li et al.). Moreover, a critical evaluation of serotonin mediation of early memory formation via 5-HT_2B_ receptor-induced glycogenolysis in the day-old chick is provided (Gibbs and Hertz). An important subject is the neuronal localization of the 5-HT_2_ receptor family in the amygdaloid complex and emotion memories (Bombardi). Modeling dysfunctional memory, mitochondrial energy metabolism in different regions of the brain and potential treatments is illustrated (Singh and Kumar). In short, the translational feature of this special issue may expand our knowledge on brain dysfunctions and inspire further investigation, thus leading to a better management of psychiatric diseases.

## Author contributions

AG wrote the editorial. AM revised and corrected the manuscript. BB worked as referee.

### Conflict of interest statement

The authors declare that the research was conducted in the absence of any commercial or financial relationships that could be construed as a potential conflict of interest.
